# Insights into discrepancies in professional identities and role models in undergraduate medical education in the context of affective burden

**DOI:** 10.3389/fpsyt.2024.1358173

**Published:** 2024-05-02

**Authors:** Rebecca Erschens, Isabelle Skrypski, Teresa Festl-Wietek, Anne Herrmann-Werner, Sophia Helen Adam, Carla Schröpel, Christoph Nikendei, Stephan Zipfel, Florian Junne

**Affiliations:** ^1^ Department of Psychosomatic Medicine and Psychotherapy, University Hospital Tübingen, University of Tübingen, Tübingen, Germany; ^2^ Tübingen Institute for Medical Education, University of Tübingen, Tübingen, Germany; ^3^ Department for General Internal Medicine and Psychosomatics, University Hospital Heidelberg, Heidelberg, Germany; ^4^ Deanery of Students’ Affairs, University’s Faculty of Medicine, Tuebingen, Germany; ^5^ Department of Psychosomatic Medicine and Psychotherapy, Otto von Guericke University Magdeburg, University Hospital Magdeburg, Magdeburg, Germany

**Keywords:** psychosomatic medicine, medical students, anxiety, depression, professional role model, professional identity

## Abstract

**Introduction:**

International evidence strongly suggests that medical students are at high risk of mental health problems. This distress, which can be mediated by a variety of individual, interpersonal and contextual factors within the curriculum, can be mitigated by effective coping strategies and interventions. Central to this discourse is the recognition that the challenges of professional identity formation can contribute significantly to medical students' distress. The focus of our study is therefore to examine discrepancies in professional identities and role models in undergraduate medical education in relation to affective burden.

**Methods:**

Medical students at different stages of university education and high school graduates intending to study medicine were surveyed in a cross-sectional study. The study employed Osgood and Hofstätter's polarity profile to evaluate the self-image of participants, the image of an ideal and real physician, and their correlation with depression (PHQ-9) and anxiety (GAD-7).

**Results:**

Out of the 1535 students recruited, 1169 (76.2%) participated in the study. Students rated their self-image as somewhere between a more critical real image of physicians and a more positive ideal image. Medical students at all training levels consistently rated the ideal image as remaining constant. Significant correlations were found between the professional role models of medical students and affective symptoms, particularly for the discrepancy between the ideal image of a physician and their self-image. Furthermore, 17% and nearly 15% reported significant symptoms of depression and anxiety, respectively.

**Discussion:**

Our study adds to the growing body of knowledge on professional identity formation in medicine and socialisation in the medical environment. The study highlights the importance of discrepancies between self-image and ideal image in the experience of depressive and anxiety symptoms. Primary prevention-oriented approaches should incorporate these findings to promote reflective competence in relation to professional role models and strengthen the resilience of upcoming physicians in medical training.

## Introduction

### Medical students’ motives, professional identity and role models

Medical students have a variety of shifting motivations for choosing to study medicine. Often, their originally altruistic and humanistic motivations are supplemented over time by scientific, craft and technological interests (1, 2) and instead focus on employment, job security, lifestyle and their families during medical school (3, 4). From an analytical or depth psychological perspective, the motivation to study medicine may be related to biographical or internal conflicts, such as early childhood experiences of illness, injury and loss. These experiences may mobilise feelings of aggression or guilt, and motivations to study medicine may be a response to or reversal of these emotions. Alternatively, the motivation may stem from fantasies of childhood salvation from physically or mentally ill parents or siblings (for an overview see Marcus (5).

International research on medical education often focuses on identifying aspects, characteristics and processes that contribute to medical students becoming well-trained physicians. For example, characteristics of the academic training itself, different admission criteria to medical school, students’ cognitive abilities, prior professional experience, general interpersonal skills, professionalism, communication skills and patient-centredness, medical-scientific competence and craftsmanship, management skills, ethical behaviour and self-reflection skills are examined (6–12). The curriculum provides the necessary knowledge and practical skills taught by health professionals, although several research studies have also examined the role of teachers as role models of professional behaviour and in the mediation of values, attitudes and professional behaviour in health care (13–15). Medical students are therefore also exposed to the values and attitudes of their fellow students, lecturers and university mentors (13, 16, 17). The socialisation process during medical education therefore plays an important role in the development of interprofessional physicians in the clinical learning environment (18–20).

As part of this socialisation process, the present study was interested in understanding more about medical students’ views and attitudes towards their own identity, their attitudes and evaluations of real and idealised images of physicians, and whether a deviation in these images and attitudes could be related to medical students’ reported affective health. In order to shed more light on this issue, a model based on three essential constructs of the physician role - the ideal and real image of the physician and the self-image of medical students - can be used to differentiate the different concepts of those roles (21). According to Koch, the term *ideal physician*, or the *ideal image* as used in this article, refers to the ideal and desired image of physicians held by a particular group associated with the medical profession (22). Those constructs indeed manifested in Koch’s results—for example, in positive characteristics attributed to physicians and high aspirations to assume the role of physician as part of an absolute and Hippocratic professional ethos. Beyond that, Koch defines the *real physician*—in this article, the “real image”—as the image of the real physician in the given contemporary context. Last, Koch refers to the attitudes and expectations of the medical profession towards itself as *self-image* (22).

Thus, the role and job profile of physicians have both positive and negative aspects that can shape the image formed of physicians amongst (pre)medical students (23). However, no consensus exists on the characteristics of the ideal physician, and research on medical students’ views of those characteristics has been limited. Across such studies, characteristics that emerged as being associated with an ideal physician have included professional competence, thoroughness, communication skills (e.g. open-mindedness, calmness and patience), empathy, warmth, ethical integrity, role security and honesty (9, 24–26).

Forming a realistic self-image is a long, difficult and usually ongoing process that helps individuals to shape their professional identity. For beginning physicians, integrating this process into their own professional role can be particularly daunting, given the high professional ethos and associated idealisation - for example, the ideal of making as few mistakes as possible. In addition to these high expectations of themselves, it is becoming increasingly clear that external and internal perceptions of the physician’s self-image are merging (27).

### Professional role perceptions using the technique of polarity profile

**Figure 1:**
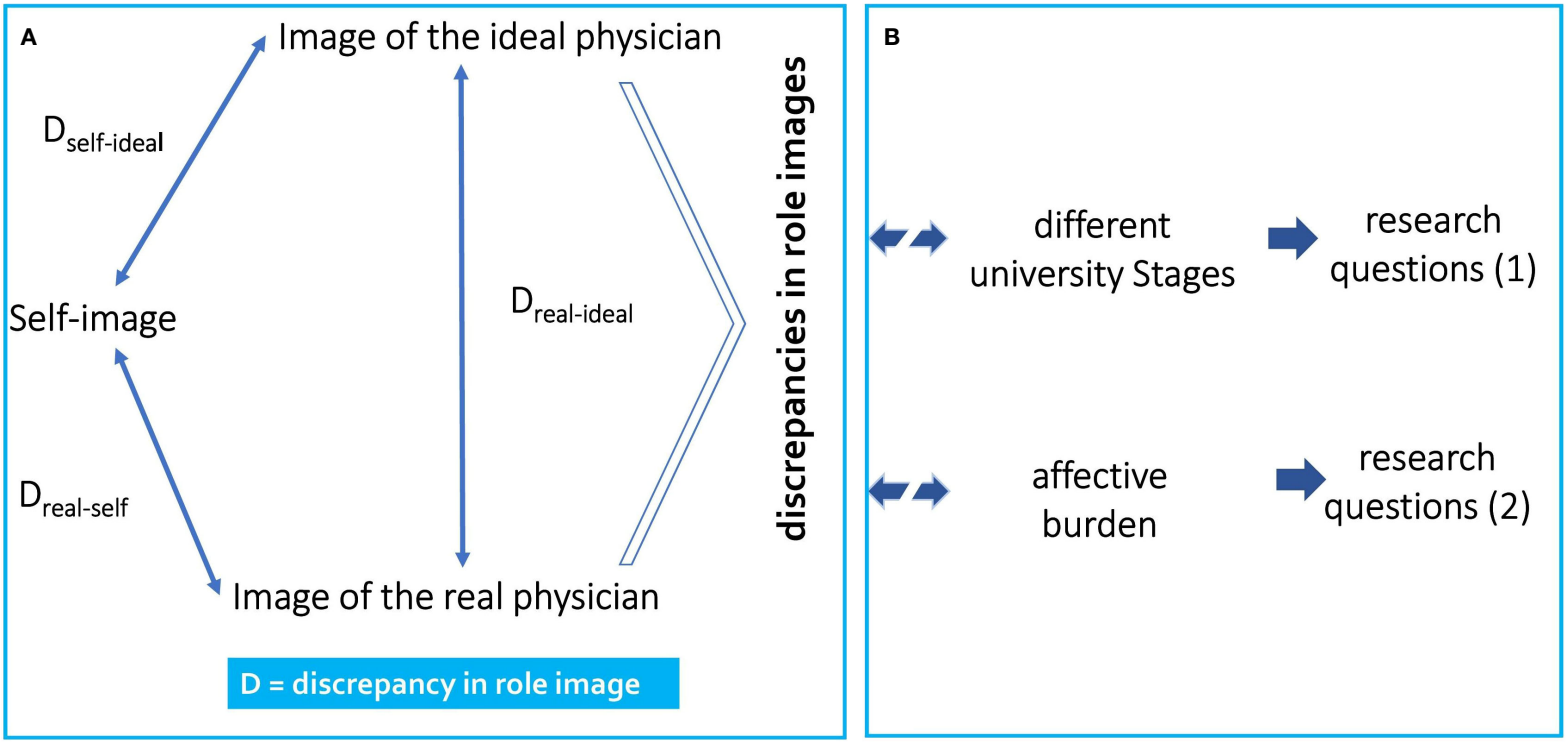
Illustration of the theoretical model underlying the present study. **(A)** The arrows indicate the three possible role discrepancies (1) self-real discrepancy, (2) self-ideal discrepancy, and (3) ideal-real discrepancy investigated in this study among medical students using the polarity profile, and **(B)** the three associations within the research questions are illustrated.

The polarity profile is a survey instrument developed by Osgood (28) and adapted by Hofstätter and Lübbert (29) for the analysis of stereotypes. In the polarity profile, associative word intensities are represented by a classification based on bipolar adjective pairs. The tool thus allows a quantitative analysis of stereotypes expressed in affective word meanings according to semantic differences. In this article we use the polarity profile based on the polarity profile revised by Speierer in 1981-1984 (30) and used by Schrauth in 2009 (21). The two author groups analysed the different role perceptions of an ideal and a real physician, as well as the self-image of a group of medical students and the general population, using different pairs of characteristics in a polarity profile. The detailed structure and procedure are explained in the methods section. The three relevant role images of the professional socialisation process are shown in **Figure 1A**.

Following the first research question, this study examines medical students and their perception of professional roles as a replication of the study of Schrauth and colleagues (21). New aspect here is that we are interested in whether there is a discrepancy between the perceived image of physicians (i.e., ideal image and real image) and the self-image of medical students at different stages of their medical education. Therefore, with Research Question 1 we investigate medical students in different phases of their university education and include a new and rather under-researched group, i.e., students interested in medical education. We hypothesise that the different degrees of exposure to the medical curriculum have an effect on the perception of physician role models.

### Psychological burden in medical students

To be sure, physicians generally occupy a prestigious position in society due to their vital role and perception of the necessity of that role, which is also reflected in the widespread popularity of the career choice (12, 23, 31). In Germany, for instance, there are now 3-5 applicants for every available spot in medical school, and the trend is rising (www.hochschulstart.de). However, after arriving at medical school, many students in their first semester become unable to cope with the increased pressure that accompanies the pursuit of their career choice, pressure that is usually caused by stressors such as academic demands, time pressure and social adjustments (32–34). Specific stressors, coping strategies and multifaceted forms of psychological distress involving risk of burnout and syndrome-related stress disorders amongst medical students are well documented in the international literature (35–40). Later, those trends amongst medical students carry over to the clinical workplace, where reduced well-being and burnout amongst physicians have also been described (e.g. 41–45).

There are recent studies showing that students or medical professionals who have (developed) a good professional identity experience less stress (e.g. 46, 47). This study here focusses on possible discrepancies between real, self- and ideal images and their association with affective burden using the technic of polarity profile.

Research question 2 examines if the possible role discrepancies are related to psychological distress. According to our hypothesis, higher discrepancies between the images are associated with higher psychological distress. We operationalized psychological distress with the report of affective symptoms, assessed with the Patient Health Questionnaire (48; PHQ module Depression and Anxiety). **Figure 1** shows the *overall Conceptual Model*.

## Material and methods

### Design, participants and procedure

The study was conducted at a university medical school in Germany using a printed and online survey. Data collection for this study was part of a larger research project within in the framework ´*Centre of excellence for the prevention of mental and psychosomatic disorders in the workplace and education Baden-Württemberg*´. This research project yielded further investigations into distinct outcome variables and differentiated subgroup and sub analyses, which have already been published elsewhere (34, 38, 49). Therefore, there may be similarities in the description of the study procedure and sample descriptions.

A total of 1,535 participants were invited from the start of a winter semester (October) until the beginning of a summer semester (April). In order to provide a differentiated analysis of the research questions, the study population consisted of two different groups of participants:

(i) A sample of N=500 high school graduates who were very interested in studying medicine. This group was invited to take part in the study during a study information event for medical studies on a paper-pencil basis;
(ii) A sample of N= 1035 medical students at five different semester stages were invited to participate:

1. n=360 preclinical students, i.e. students in the 1^st^ and 3^rd^ semesters, each with n=180.

2. n=370 medical students in the clinical section, with students in the 6^th^ with n= 180 and students in the 9^th^ semesters with n=190.

3. n=305 final-year medical students completing their practical year.

Data collection of medical students was spread over the survey period in small groups. Medical students in the 1^st^, 3^rd^, 6^th^ and 9^th^ semester completed a paper version of the questionnaire made available. Medical students in their final year of training were recruited using an online version of the questionnaire, due to the wide geographical distribution of the clinics where students undertake their practical training. All medical students in the respective semester were invited to participate. No specific inclusion criteria were used. Medical students preparing for state examinations (e.g. 4^th^ and 10^th^ semesters) were excluded to minimise inflated stress rates. All participants who provided their written consent were invited to participate and included in the study analysis.

### Structure of university medical education in Germany

The traditional curriculum for human medicine in Germany is divided into a pre-clinical (1^st^-4^th^ semester) and a clinical study phase (5^th^-12^th^ semester), with a standard study period of 6 years. The pre-clinical part of the programme includes scientific subjects, a dissection course and communication courses. The first state medical examination is followed by a four-month clinical internship to familiarise students with medical patient care. In the clinical part of the programme, students learn about diseases, their pathogenesis and treatment in more than 20 clinical and cross-sectional subjects. The written second state examination is followed by a twelve-month practical year, which ends with the (oral) third medical examination. The aim of the practical year is to put the knowledge and skills acquired during the course into practice by carrying out all aspects of diagnosis, therapy and patient care under the guidance of a supervising physician. For a detail overview see (50, 51). The medical curriculum has been reformed with the “Masterplan 2020 for Medical Students”. The aim of Masterplan 2020 is to strengthen the scientific character and practical relevance of medical training see (52, 53).

### Measurements

The scope of the questionnaire encompassed participants’ demographic background, the polarity profile and the Patient Health Questionnaire (PHQ; 48).

#### Demographics

The questionnaire dimensions included demographic information: their age, gender, stage of medical training, marital status, and whether they had graduated in Germany or in another country (For more information please see (34). High-school graduates were also asked whether they were interested in studying human medicine only or interested in other subjects as well.

#### Polarity profile

The polarity profile is a survey instrument used in empirical social research and is originally developed and validated by Osgood (28), adapted by Hofstätter and Lübbert (29). For this study the polarity profile revised by Speierer in 1984 (30) and used by Schrauth in 2009 (21). The polarity profile is considered to be an approved instrument by the aforementioned authors and is available in the German language. For this manuscript, the instrument was translated (back and forth) by a bilingual medical physician.

The polarity profile consists of dimensions formed by 18 bipolar adjective pairs (e.g. “weak vs. confident”), which are rated on a 7-point Likert scale from 1 (i.e. the extreme left pole) to 7 (i.e. the extreme right pole), with 4 being “neutral”. The participants are asked to create a polarity profile for three mental images that are important for the process of professional socialisation: a self-image (“as I really am”), an image of physicians they know (“according to my experiences with physicians”) and an ideal image of a physician (“according to my ideas of an ideal physician”). To compare the polarity profiles, the semantic *distance D* indicates how far apart the profiles are metrically (cf. Schrauth et al., 21).

#### Patient health questionnaire

For this study, the German translation and validation of the Patient Health Questionnaire (PHQ; 48) carried out (see 54–56). Please note that the PHQ data were only collected from medical students in semesters 1, 3, 6 and 9 or in their final year of study, due to the administrative requirements and restrictions and time available for data collection from high school graduates. Thus, only a shortened version of the questionnaire was possible for us to use.

##### GAD-7

Anxiety was assessed with the General Anxiety Disorder (GAD-7) module of the Patient Health Questionnaire (PHQ), a self-report questionnaire for screening generalised anxiety disorder. The GAD-7 consists of seven items to be rated on a 4-point scale ranging from 0 (*not at all*) to 3 (*almost every day*) for a sum score from 0 to 21, with scores of ≥5, ≥10 and ≥15 indicating symptoms of mild, moderate and severe anxiety, respectively. The GAD-7 has a Cronbach’s alpha of.89.

##### PHQ-9

Symptoms of depression were assessed using the module PHQ-9 (55), whose items were rated on a 4-point scale ranging from 0 (*not at all*) to 3 (*almost every day*) for a sum score from 0 to 27, with scores of ≥5, ≥10 and ≥15 indicating symptoms of mild, moderate and severe depression, respectively. Cronbach’s alpha was.89.

### Statistical analysis

Statistical analyses were performed with IBM’s SPSS for Windows version 27.0. For descriptive summary statistics, total sum scores, mean values, median values and standard deviations for the validated instruments were calculated. Due to the psychometric properties of data from the PHQ (i.e. bias to the left for healthy) and because normal distribution was violated, semantic distances in non-parametric tests such as the Kruskal–Wallis test and the Mann–Whitney *U* test were calculated. Pearson or Kendall’s tau was determined correlation methods were used to calculate correlative associations between the individual constructs and control variables. Replace missing values, multiple data imputation was applied (57). Last, to analyse the role models and images of the ideal physician, real physician and self-image as well as their semantic distances (*D*), standard deviations (*SD*) were calculated for the respective mean (*M*) values.

## Results

### Sample description and demographics

Of the 1535 students recruited, 1169 (76.2%) participated in our cross-sectional study. The average age of the medical students was 24.87 years (*SD*=4.38). Almost 67% of the participants described themselves as women. More than half (>58%) were in an active partnership, and more than a third had a part-time job. About 40% stated that they had professional pre-qualifications, whether in medical-related training or from a general study programme, and more than 20% reported that their parents were physicians themselves. Approximately 6% of medical students stated that they had completed their A-levels in a country other than Germany. **Table 1** presents the demographic data.

**Table 1:** Results of demographic data.

Variable	psychometric
***N (*Invited/processed)**	1535/1169
**RR (%)**	76,2%
Semester	
**High School graduates**	n=500/n = 423
**1**	n=180/n = 149
**3**	n=180/n = 143
**6**	n=180/n = 123
**9**	n=190/n = 154
**final year**	n=305/n = 177
age [years]	
**High School graduates**	M=18,24; SD=2,69
**Medical students**	M=24,87; SD=4,38
gender (%)	
**female**	66.9
**male**	33.2
**diverse**	n.a.
prior vocational training^a^ (%)	
**yes**	40.7
In relationship (%)	
**yes**	58.8
Side job (%)	
**yes**	36,2
Parents medically employed	
**yes**	20.9

^a’^Did you start/complete professional training or studies before studying medicine?’ n.a, not applicable.

### Professional role perceptions

**Figure 2:**
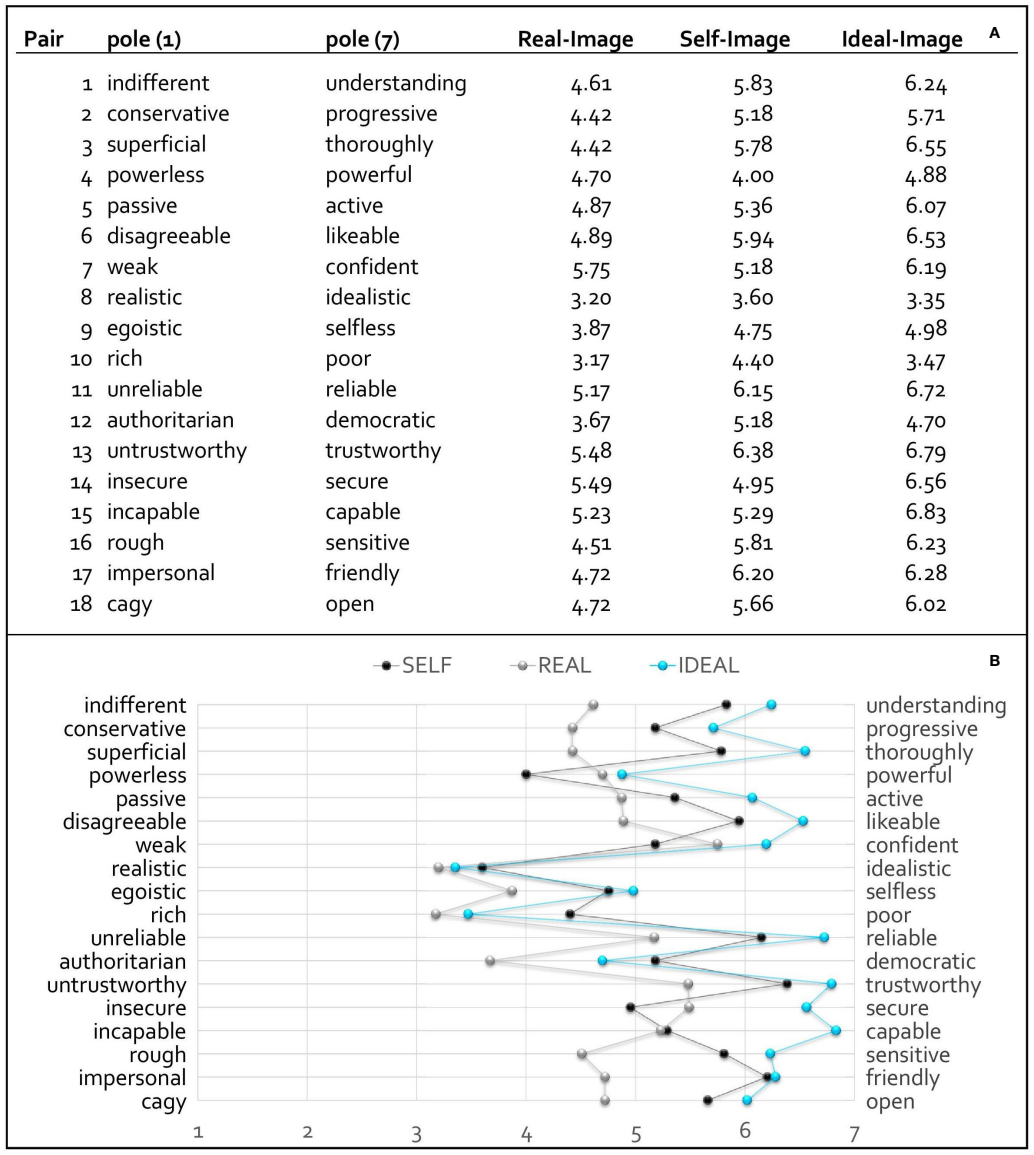
Illustration of the numerical **(A)** with mean values and graphical representation **(B)** of the three images: real image, self-image and ideal image with polarity profile.

The self-image of all subgroups ranged between the real image (i.e. closer to the left pole) and the ideal image (i.e. closer to the right pole) of physicians for nearly all adjective pairs. On average, the self-image of the individual groups and of the entire sample was closer to the ideal image than to the real image, both with the pairs of adjectives considered individually and when totalled. **Figure 2A** shows the numerical representation and **Figure 2B** the graphical representation of the three images: real-image, self-image and ideal image.

For instance, participants tended to describe the real image of physicians as “realistic”, “rich”, “authoritarian”, “confident”, “capable” “trustworthy” and “secure” (*M* = 3.17–5.75). In the perception of their own image, the students tended to describe themselves as “understanding”, “thorough”, “likeable”, “reliable”, trustworthy” and “friendly” (*M* = 5.78–6.38). The respondents’ assessment of the ideal image showed the strongest assessments for the adjectives of the right-hand pole except for “realistic” versus “idealistic”, “rich” versus “poor” and “authoritarian” versus “democratic”.

### Professional role perceptions by subgroups

**Table 2** details the individual-level results for the assessment of the different images and the distances between them in the different subgroups. A Mann–Whitney *U* test revealed specific differences between the different images. Results of the Kruskal–Wallis test for all three images and distances showed significant between-group differences except regarding the ideal image (real image: χ^2 ^= 137.6, *df* = 5, *p* <.001; self-image: χ^2^ = 54.93.74, *df* = 5, *p* <.001; D_self–real_: χ^2^ = 66.7, *df* = 5, *p* <.001; D_self–ideal_: χ^2^ = 61.6, *df* = 5, *p* <.001; D_ideal–real_: χ^2^ = 104.3, *df* = 5, *p* <.001). No between-group difference emerged concerning the ideal image (χ^2^ = 0.51, *df* = 5, *p* =.992).

**Table 2:** Exact overview among single results on the evaluation of the role models.

Dimension	Mean value (SD)	High school graduates	Freshmen	3^rd^ semester	6^th^ semester	9^th^ semester	Final year	Total sample	p
**Self-image**	M(SD)	5.44 (0.46)	5.24 (0.53)	5.20 (0.50)	5.27 (0.55)	5.17 (0.51)	5.28 (0.60)	5.31 (0.52)	<0.01**
**Ideal-image**	M(SD)	5.80 (0.38)	5.78 (0.50)	5.77 (0.40)	5.80 (0.43)	5.77 (0.44)	5.76 (0.52)	5.78 (0.43)	n. s.
**Real-Image**	M(SD)	4.90 (0.65)	4.36 (0.62)	4.49 (0.63)	4.59 (0.65)	4.35 (0.59)	4.43 (0.77)	4.60 (0.69)	<0.01**
**D_real-self_**	M(SD)	4.63 (0.99)	5.24 (0.95)	5.03 (1.15)	5.02 (0.97)	5.10 (0.88)	5.12 (1.19)	4.93 (1.05)	<0.01**
**D_self-ideal_**	M(SD)	3.98 (0.80)	4.49 (0.90)	4.28 (0.89)	4.30 (0.94)	4.38 (0.89)	4.33 (0.95)	4.22 (0.89)	<0.01**
**D_real-ideal_**	M(SD)	4.64 (1.12)	5.46 (1.05)	5.26 (1.12)	5.14 (1.12)	5.44 (0.95)	5.33 (1.22)	5.08 (1.16)	<0.01**

^**^Highly significant; n.s., not significant.

**Figure 3:**
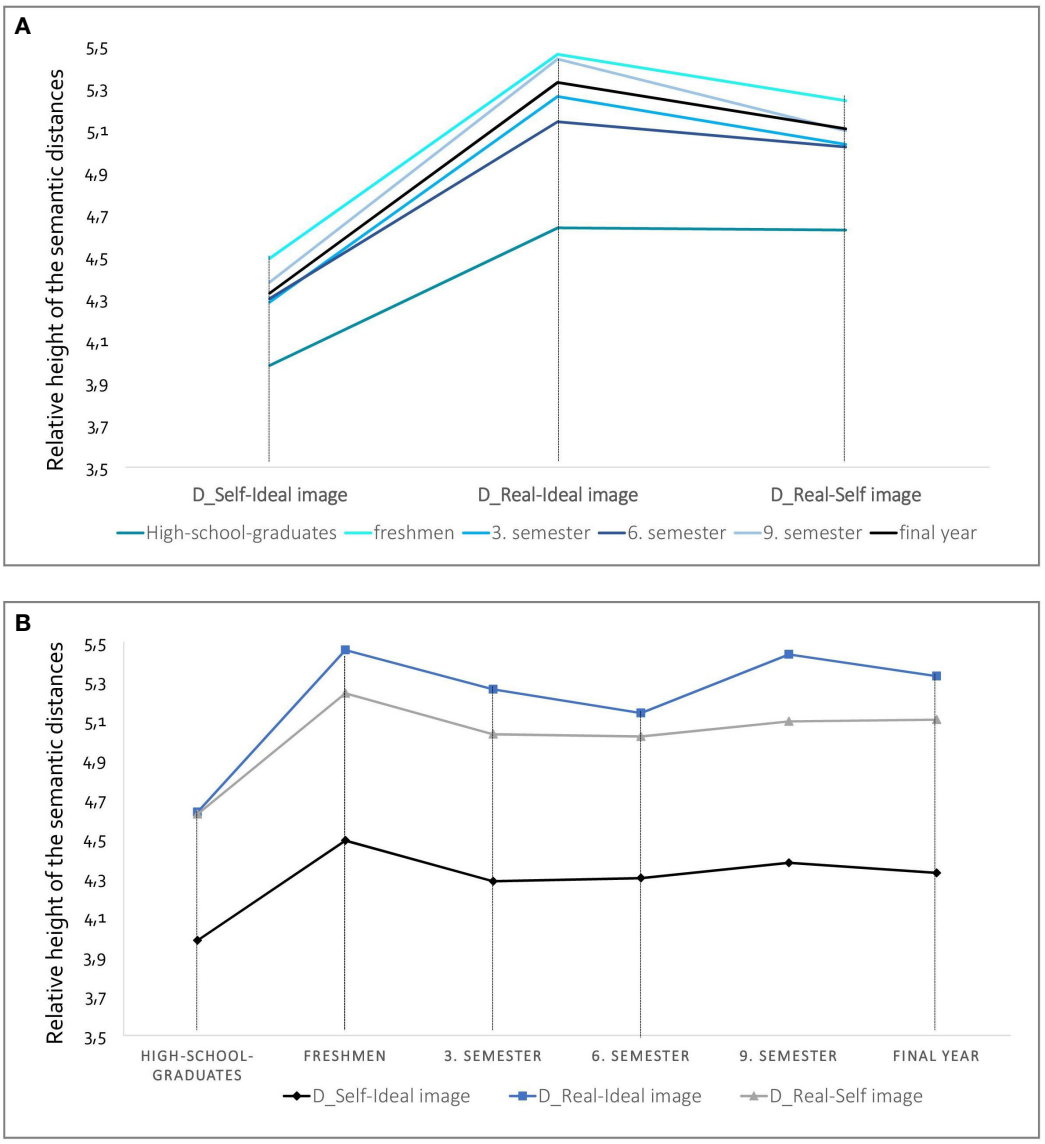
Illustration of the semantic distances to the respondents’ ideas about the real and ideal image of the physician and their self-image at the time of the different training stages. In **(A)** the discrepancies between *Self-Ideal*, *Real-Ideal* and *Real-Self image* are plotted on the x-axis. In **(B)** the different training stages are plotted on the x-axis. The presentation of the lines is not a chronological progression, but rather cross-sectional data collection within the respective subgroup studied.

**Figures 3A, B** illustrate the differences for the three distances D_real–self_, D_self–ideal_ and D_real–ideal_ in the six subgroups. Whether by trend or on average, medical students in all semesters demonstrated the same pattern: the greatest distances resulted from the *real–ideal distance*, followed by real–self distance. The divergence of *self–ideal had the smallest* distances. For all three distances, high school graduates had the least numerical distances, and medical students in Semester 1 had the greatest. Differences between students in Semesters 3, 6 and 9 and their final year emerged only for D_real–ideal_ Amongst high school graduates, there was no difference within the real–ideal and real–self distances. **Supplementary Table 1** summarises all correlations and Cronbach’s alpha values for reliability.

### Affective burden

**Table 3** reports the results on GAD-7 and PHQ-9 amongst the six subgroups. Mean scores in the sample were *M* = 5.79 (*SD =* 4.59) for the PHQ-9 and *M* = 5.58 (*SD =* 3.96) for the GAD-7. Results showed significant between-group differences for all outcomes (PHQ-9: χ^2^ = 27.32, *df* = 4, *p <.*001; GAD-7: χ^2^ = 24.96, *df* = 4, *p <.*001). A Mann–Whitney *U* test revealed significant differences in symptoms of anxiety and depression. Students in Semester 3 had significantly higher scores for symptoms of depression (*M* = 6.96, *SD* = 4.24) and symptoms of anxiety (*M* = 7.01, *SD* = 4.28), followed by students in Semester 9. More than 17% (*n =* 128) and nearly 15% (*n =* 111) of participants reported significant symptoms of depression and anxiety, respectively. Again, students in Semester 3 (*n =* 37, 26.6% for PHQ-9; *n =* 25, 17.5% for GAD-7) and Semester 9 (*n =* 29, 19.0% for PHQ-9; *n =* 25, 16.2% for GAD-7) were more stressed than students at all other stages of medical education.

**Table 3:** Overview among the results of GAD-7 and PHQ-9 within subgroups.

Dimension	Psycho-metrics	Freshmen	3^rd^ semester	6^th^ semester	9^th^ semester	Final year	Total sample	p
**PHQ-9**	M(SD)	5.03(4.21)	7.01(4.28)	5.65(4.57)	5.67(4.47)	5.60(5.01)	5.79(4.59)	<0.01**
	**PHQ sum score ≥10**	n=17; 11,4%	n=37; 26.6%	n=17; 13.8%	n=29; 19.0%	n=28; 15.8%	n=128; 17.3%	<0.01**
**GAD-7**	M(SD)	5.14(3.63)	6.52(3.83)	5.71(4.32)	5.74(3.75)	4.95(4.14)	5.58 (3.96)	<0.01**
	**GAD-7 sum score ≥10**	n=20; 13.4%	n=25; 17.5%	n=19; 15.4%	n=25; 16.2%	n=22; 12.4%	n=111; 14.9%	<0.01**

^**^Highly significant.

### Associations between role images and GAD-7 and PHQ-9 scores

**Figure 4:**
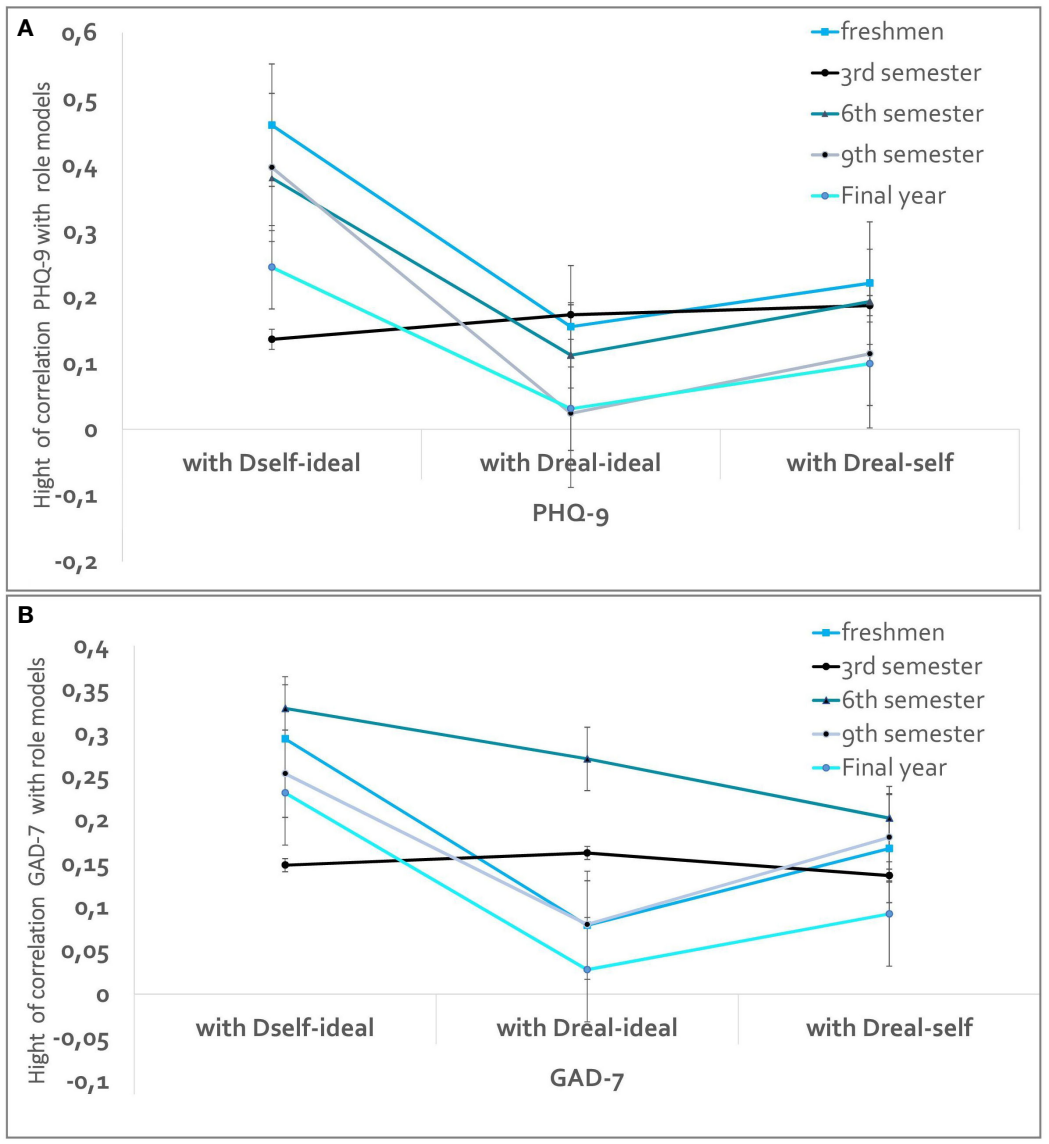
Illustration of the correlations of the distances with PHQ **(A)** for PHQ-9 & **(B)** for GAD-7). The discrepancies between *Self-Ideal*, *Real-Ideal* and *Real-Self image* are plotted on the x-axis. The different training stages are plotted as lines that present not a chronological progression, but rather cross-sectional data collection within the respective subgroup studied.

There is a clear trend across the sample: The strongest correlation was found between the divergence of the self-image from the ideal image (D_self-ideal_) for the GAD-7 (*r* = .242, *p* <.01) and the PHQ-9 (*r* = .309, *p* <.01). **Figures 4A, B** shows the correlations between the individual images and the discrepancies with the secondary parameters of the PHQ-9 (a) and GAD-7 (b) scores for all subgroups. With the exception of the 3^rd^ semester in figure (a) and the 6^th^ semester in figure (b), all other groups show a similar trend. The highest correlations were found between the divergence of the self-image and the ideal image (D_self-ideal_), while the lowest correlations were found between the real image of the physician and the ideal image with affective burden. The trend shows that the 3^rd^ semester has the highest correlation with the divergence between the real and ideal image of the physician, as well as with the PHQ-9 and the GAD-7. For students in the 6th semester, all discrepancies in the trend seem to be associated with anxiety. **Supplementary Table 2** provides information on the individual correlations.

## Discussion

### Role models and discrepancies between stages of university medical education

The results concerning our *first research* question indicated that self-image overall and in all subgroups for nearly all pairs of adjectives was rated between the real image (i.e. closer to the left pole) and the ideal image (i.e. closer to the right pole). The real image of physicians tended to be described as “realistic”, “rich”, “authoritarian”, “self-confident”, “reliable”, “trustworthy” and “secure”. Meanwhile, regarding self-image, the participants tended to describe themselves as “understanding”, “thoroughly”, “sympathetic”, “reliable”, “trustworthy” and “friendly”. Last, the ideal image of physicians was predominantly described with the adjectives “understanding”, “thoroughly”, “likeable”, “reliable”, “trustworthy”, “capable” and “secure”.

The results differed significantly across the subgroups except regarding the *ideal image* of physicians, which was rated similarly by students at all stages of medical education, including the high school graduates. The semantic distances between the self- versus real images, real versus ideal images and self- versus ideal images all revealed that the high school graduates had the significantly shortest distances between the images and that the first-year students tended to have the greatest distances. Last, all groups showed a similar pattern, with the largest semantic distance appearing in the discrepancy between the real and ideal images and the smallest for the discrepancy between self- and ideal images.

Our findings for the group of medical students can be compared with the results of benchmark studies carried out in the 1980s (see Speierer et al.) and in 2009 by Schrauth et al. (21, 30), using the polarity profile technic. Here, too, the medical students showed a differentiated perception of images of physicians and of themselves. The order or pattern of the different images (i.e. real image, self-image and ideal image) did not change significantly, so that there was a surprising consistency in the characterisation of the real and ideal image of physicians, especially in the ideal image.

Other findings included that the self-image in our study continued the tendency observed in the cited studies to lie between the real image of a physician (more to the left pole) and the ideal image (more to the right pole). The students surveyed by Speierer et al. (30) attributed more activity to the real image of a physician than to their own self-image, whereas our study and that of Schrauth et al. (21) shows that medical students perceive themselves as more active and today’s physicians as more passive than their former fellow students. The perspective on the adjective pair “rich” and “poor” has also shifted in recent years. Whereas the medical students surveyed in Speierer’s study in 1984 (30) considered the ideal image of a physician to be as rich as possible, our respondents, similar to Schrauth et al. (21), considered this to be less desirable and even described the real image of physicians as richer than the ideal. Another change in perspective manifested in the adjective pair “incapable” and “capable”. Whereas our participants rated themselves as being more capable than real physicians as they perceived them, the participants in the two prior studies perceived real physicians as being more capable than themselves.

Gilligan et al. (58) conducted a similar study on professional role models amongst medical students in their first semester and final year of medical school in Germany and Australia. Those authors found that perceptions of the ideal physician were common to all respondents, whereas perceptions of real physicians and self-image differed by country and cohort, particularly in perceptions of “confidence”, “strength”, “skills” and “safety”.

### Suggestions for ranking differences according to role models and semester groups

The similarity between medical students in different semesters and prospective students in in their assessment of the *ideal image* of a physician can be attributed to several possible causes. One being the homogeneity in societal ideas about what constitutes an ‘ideal physician’ that is shared by students. These general ideas could be widespread in society, medical education, and the media, and may serve as a reference for students’ ideal image. Medical education may influence perceptions of the ideal physician, as curricula and clinical experiences may promote certain characteristics and qualities (13). Additionally, social norms and expectations may play a role, as medical professions are highly valued by society and certain expectations are placed on physicians (for an overview see 23, 59) Furthermore, it is possible that the self-selection of students interested in studying medicine may result in individuals with pre-existing personality traits and values that align with the typical ideal-image of a physician being admitted into the programme (12). Students at all levels demonstrate a small discrepancy between their self-image and the ideal image of a physician. One possible explanation is strong professional identification (see, for example, the theory of professional identity (60), as students who choose to study medicine are already confident in their abilities and have the characteristics that match the professional profile of a physician. Another factor may be self-selection: Individuals interested in studying medicine often have personality traits and values that match the idealised image of a physician (61). In addition, students can actively cultivate these desirable characteristics through professional development, leading to a convergence of their self-image with the ideal image.

Moreover, high school graduates interested in studying medicine have similar attitudes towards images of physicians as those already enrolled in medical school, however they show the smallest discrepancies for the assessments of real physicians versus ideal physicians, ideal self-image versus real physicians, and self-image versus ideal physicians. These differences may be attributed to various factors. On one hand, students often strongly identify with the physician’s job description, which aligns with their self-image. Targeted preparation and information gathering about the profession can help develop more realistic expectations. These students focus on their career development early on and work to develop the necessary traits. Their strong motivation for self-improvement contributes to their active pursuit of personal and professional development, resulting in fewer discrepancies between their different images. This is supported by *Atkinson’s motive theories* (62) and the *self-determination theory* of Deci and Ryan (63).

The significant differences in freshmen medical students’ ratings of role models and their self-image can be attributed to several psychological processes. The challenges of commencing medical school may result in insecurities and adjustment processes that make it harder to reconcile one’s expectations with reality (38, 64, 65). In their first semester, students studying medicine may experience a dissonance between their idealised perceptions of physicians and the realities of their studies. This could lead to a reassessment of their attitudes and expectations. Festinger’s *cognitive dissonance theory* (66) may explain this phenomenon.

### Affective burden in medical education in different stages of medical education

The second research question investigated whether there is a correlation between the role discrepancies mentioned earlier (self-image vs. real-image, real-image vs. ideal-image, and self-image vs. ideal-image) and the severity of symptoms of depression and anxiety.

Students in the third semester had significantly higher scores for symptoms of depression and anxiety than the other participants, followed by students in the ninth semester. A possible interpretation of the affective peaks in the 3^rd^ and 9^th^ semesters may lie in the specific requirements of the medical curriculum. In the third semester, medical students in this study have a very full timetable with very different longitudinal courses such as communication and scientific work, natural sciences (e.g. physiology, molecular biology, physics, anatomy, chemistry) and the dissection course. For medical students, this first contact and work with cadavers can be stressful and anxiety-provoking (see e.g. 67, 68). The ninth semester curriculum of this sample contained a high proportion of examination courses with patient contact. Physician-patient communication in the context of “illness, suffering and dying” may be perceived as stressful by students (see e.g. 34, 69, 70).

Meta-analyses and relevant studies have shown that clinically relevant depression affects approximately 5-48% of medical students at various stages worldwide (39, 40, 71). Our results contribute to the international literature on affective burden in medical students in various ways, although they tend to be at the lower end of the wide range: In total, 17% (n = 128) of participants reported significant symptoms of depression, while almost 15% (n = 111) reported significant symptoms of anxiety. On the one hand, there may be methodological reasons behind these differences. These include the considerable heterogeneity in international reporting of prevalence rates of anxiety and depression symptoms among medical students, e.g. different instruments, interpretation and use of cut-off values, etc. On the other hand, specific contextual factors may include various aspects that influence the study situation and life circumstances of students (for an overview see 72). For example, these include differences in study settings and demands, such as curricula, examinations. In addition, students’ well-being may be influenced by the quality of the learning environment, including faculty support and availability of resources. Furthermore, the social environment, including family support, friendships, social integration and interaction with fellow students, as well as individual attributes such as age, gender, ethnicity, socio-economic status and previous experiences. Researchers have highlighted the benefits of functional coping strategies for medical students, including positive framing, social support from family and friends, resilience building and exercise, active stress management training and early offers of support (38, 73–75).

### Association of role perceptions with affective burden

The study found a moderate but significant correlation between the self-image and ideal image discrepancy of physicians and increased levels of anxiety (GAD-7) and depression (PHQ-9) in medical students. This correlation can be explained by various psychology theories, such as perfectionism approaches. The perfectionism cognitive theory of Flett, Hewitt, Blankstein and Gray (76) might explain the discrepancy between the self-image and the ideal image by the fact that medical students set high personal standards for themselves and evaluate themselves self-critically, especially when they feel that they are not living up to these standards. This self-critical thinking and fear of failure, often associated with perfectionism, could lead to increased levels of anxiety and depression (77, 78).

The discrepancy between self-image and ideal image and the increased levels of anxiety and depression among medical students could also be explained in more detail by possible professional identity insecurity, based on Erik Erikson’s work on the model of identity development (79). According to Erikson, individuals pass through different stages of identity development throughout their lives. In the case of medical students, who are at a crucial stage in their professional development, a discrepancy between their self-image and the ideal image of a physician may lead to uncertainty about their future professional role. This uncertainty may result from an inability to reconcile their own professional goals and expectations with the ideal image of a physician. Students may wonder whether they are up to the demands of the profession or whether they can achieve their professional goals. This uncertainty and related identity issues can lead to psychological distress such as anxiety and depression.

Albert Bandura’s self-efficacy theory (80, 81) provides a useful perspective for understanding the beliefs of medical students (82). This model states that a person’s belief that they can successfully perform certain actions influences their motivation and ability to overcome challenges. Self-efficacy beliefs in medical students are related to motivation and performance (e.g. 83) and greater resilience to psychological distress (e.g. 84, 85). Self-efficacy may play a role in the discrepancy between the self-image and the ideal image of physicians: When medical students feel that they do not fit the ideal image of a physician, it can affect their confidence in their ability to succeed and achieve their goals. This lack of self-efficacy can lead to increased anxiety and depression as students feel overwhelmed and not up to the task. However, it is important to note that other factors may also play a role that were not considered in this study. Additional research is warranted to better understand these relationships and identify possible intervening variables.

The lower correlations between the rather high discrepancies between real and ideal images with anxiety and depression, compared to the other discrepancies could also be explained by Erikson’s psycho-social theory (86). According to Erikson’s theory, individuals go through different stages in their development, each of which is characterised by a central psychosocial crisis. The phase of intimacy and solidarity versus isolation occurs in young adulthood. During this stage, individuals seek to develop close and trusting relationships with others, both personally and professionally. Medical students at this stage of their development may be more concerned with consolidating their professional identity as future physicians and building relationships with colleagues and mentors. As a result, discrepancies between their ideal image of a physician and the actual physician they experience may have less impact on their mental health, as they are at a stage where the focus is on building stable relationships rather than comparing themselves to idealised notions. During training, students should learn to adapt to the realities of the medical profession and adjust their expectations accordingly. A flexible approach to the challenges and demands of the profession can help to reduce emotional stress.

The discrepancy between real and ideal-images as well as between self-images and real-images was less associated with psychological stress for the entire cohort. One exception was a ceiling effect among students in the sixth semester with regard to the correlation between real and ideal-images and the experience of anxiety. The demanding and comprehensive curriculum, with many internal medicine-focused examination courses, can put students under enormous time pressure, which may lead them to rush through subjects rather than study them in depth. The discrepancy between their ideal of a physician and the professional reality could therefore be exacerbated by a rather superficial study of the clinical subjects or by their own “choice of specialisation”. In addition, the perceived academic pressure and the examinations in the semester can lead to students spending a large part of their time and energy trying to fulfil the requirements. Furthermore, this perceived stress or (exam) anxiety could be analysed as a transfer of the real physician-object relationship to the contact and interaction with the available physician lecturers (87, 88). The Objective Structured Clinical Examination (OSCE), introduced by Harden et al. (89) and implemented in medical school of this cohort at the end of the 6^th^ semester is an important examination in which students have to demonstrate their clinical skills. The high pressure to perform and the intensive preparation (90) for this practical examination format which can lead students to invest a lot of time and energy in preparing for the exam. This may leave less room for reflection on their own professional identity and the development of a realistic self-image and ideal of the physician. The stress associated with the OSCE could therefore lead to a greater discrepancy between the ideal and the real image.

Finally, with the exception of high school graduates and freshmen (see above), we could not establish a specific pattern or a global assignment of the various discrepancies to the individual semester stages, nor any additional associations with affective symptoms. Nevertheless, associations with transitions or focal themes within the curriculum are conceivable, for instance through intensive courses in basic and natural sciences, or the dissection course in the pre-clinical section or examination. Further influences might also include additional theoretical learning, communication and practical skills training in the clinical section, increased contact with real patients or continued development of craftsmanship (e.g., first surgical experience during the practical year). Other aspects that could be included in a more detailed discussion might be the alternating phases of identification with patients, fantasies related to helping and healing, various defence mechanisms, etc. (for an overview see 2, 5).

### Limitations

Amongst our study’s limitations, its cross-sectional design limited the causal relationships between the images of physicians and symptoms of depression and anxiety to being examined only in explorative ways with associative statistical methods and thus prevent their causality from being sufficiently understood. Future longitudinal studies with similar cohorts are therefore needed to further investigate those underlying causal relationships. Furthermore, in addition to quantitative questionnaires, researchers should evaluate data from focus groups.

We also recommend additional individual-level variables for future research to gain a more comprehensive understanding of the relationship between medical students’ role models and images of physicians and their psychological well-being. In particular, personal coping strategies, personality traits such as perfectionism or neuroticism, previous professional experience, expectations and motivations for a career in medicine and affiliation with minority groups should be explored in more detail.

### Conclusion and implications

This study presents findings that contribute to the growing body of knowledge on professional identity formation in medicine and socialisation in medical education. Medical schools serve as centres for teaching and learning, and as spheres of influence that shape students’ strategies, attitudes, and mindsets. The development and conceptualisation of medical students’ professional identity has significant implications for their mental health, use of competencies and skills, experiences of empathy, and relationships with colleagues and patients.

University training should include reflection on one’s own ideas, expectations, and perceptions regarding self-image to develop professional identity. Additionally, related dimensions such as resilience factors and the experience of stress and affective burden should be addressed and reflected upon. A wide range of health literacy programmes at medical faculties could include topics such as healthy sleep, exercise, problem-solving, dealing with perfectionist tendencies, self-compassion, mindfulness, and non-pharmacological interventions. The formal teaching of medical practice covers the necessary knowledge, skills, and behaviours. Additionally, students are taught compassion and respect for patients at the bedside. This process is reinforced by positive role models and faculty, which help to shape students’ character and values. Simultaneously, they facilitate a global process of identity formation (14, 15, 20, 91). Role models in medical studies aid students’ implicit learning processes and convey implicit behavioural norms. In addition to teachers, individuals in the immediate environment, such as fellow students, can also communicate significant unofficial learning content and assist others in becoming familiar with the role of a medical student (13, 92).

## Author’s note

IS conducted a baseline analysis of the polarity profile with the populations studied here, focusing on self-efficacy and sense of coherence and a historical comparison, which was included in her German-language dissertation (49).

## Data availability statement

The authors of the study received permission from the Medical Faculty of the University and University Hospital to collect and process the data with the restriction that the data may not be made publicly available due to its confidential nature. The original contributions presented in the study are included in the article and in the **Supplementary Material**. Further inquiries can be directed to the corresponding author.

## Ethics statement

The study was approved by the ethics committee of the University Hospital and Medical Faculty of the University of the tertiary hospital (number 053/2014BO1) and was carried out in accordance with the recommendations of the ICH-GCP guidelines, Declaration of Helsinki in its current version (Fortaleza 2013). All subjects gave written informed consent in accordance with the Declaration of Helsinki. The studies were conducted in accordance with the local legislation and institutional requirements.

## Author contributions

RE: Conceptualization, Data curation, Formal analysis, Investigation, Methodology, Software, Validation, Writing – original draft, Writing – review & editing. IS: Data curation, Formal analysis, Writing – review & editing. TF-W: Data curation, Formal analysis, Writing – review & editing. AH-W: Conceptualization, Supervision, Validation, Writing – review & editing. SA: Methodology, Validation, Writing – review & editing. CS: Formal analysis, Validation, Writing – review & editing. CN: Conceptualization, Funding acquisition, Investigation, Supervision, Validation, Writing – review & editing. SZ: Funding acquisition, Methodology, Resources, Supervision, Validation, Visualization, Writing – review & editing. FJ: Conceptualization, Investigation, Project administration, Resources, Supervision, Validation, Visualization, Writing – review & editing.
