# Supplementary material for: Insights into discrepancies in professional identities and role models in undergraduate medical education in the context of affective burden
Source: Front Psychiatry. 2024 May 2;15:1358173. doi: 10.3389/fpsyt.2024.1358173 (PMC11097199; doi:10.3389/fpsyt.2024.1358173)
Supplement: Supplementary file 1 [file Table_1.docx]

Supplementary Material

Insights into discrepancies in professional identities and role models in undergraduate medical education in the context of affective burden

Rebecca Erschens, Isabelle Skrypsk, Teresa Festl-Wietek, Anne Herrmann-Werner,

Sophia Helen Adam, Carla Schröpel, Christoph Nikendei, Stephan Zipfel & Florian Junne

*** Correspondence:** Corresponding Author: [rebecca.erschens@med.uni-tuebingen.de](mailto:rebecca.erschens@med.uni-tuebingen.de)

# Supplementary Table 1

Correlation of Professional Role Models with GAD-7 and PHQ-9

|  | PHQ-9 | GAD-7 | Self-image | Real-Image | Ideal-image | D _self-ideal_ | D _real-ideal_ | D _real-self_ |
| --- | --- | --- | --- | --- | --- | --- | --- | --- |
| PHQ-9 | .82^a^ |  |  |  |  |  |  |  |
| GAD-7 | .708** | .82 |  |  |  |  |  |  |
| Self-image | -.283** | -.206** | .76 |  |  |  |  |  |
| Real-Image | -.084* | -.101** | .291** | .86 |  |  |  |  |
| Ideal-image | -.099** | -.033 | .481** | .275** | .75 |  |  |  |
| D _self-ideal_ | .309** | .242** | n.a^b^. | n.a. | n.a. | n.a. |  |  |
| D _real-ideal_ | .081* | .113** | n.a. | n.a. | n.a. | n.a. | n.a. |  |
| D _real-self_ | .142* | .138** | n.a.. | n.a.. | n.a.. | n.a. | n.a. | n.a. |

**d**

**e**

**f**

*Note:* The Pearson correlation coefficient was used between two continuous variables; if one of the two metric variables was not normally distributed, Kendall's tau was determined; * indicates *p*<.05, ** indicates *p*<.01, *** indicates *p*<.001; *r* = .10 indicates a small, *r* = .30 a medium and *r* = .50 a large effect (Cohen, 1988); ^a^ *Cronbach's alpha in the diagonal; ^b^Not tested due to content reasons.*

# Supplementary Table 2

**Reporting of information on the individual correlations.**

| Subgroups | Semantic distances | Correlations | |
| --- | --- | --- | --- |
|  |  | GAD-7 | PHQ-9 |
| Freshmen | D_self-ideal_  D_real-ideal_  D_real-self_ | r= 0.293**  r= 0.079  r= 0.167* | r= 0.460**  r= 0.155  r= 0.221** |
| 3^rd^ semester | D_self-ideal_  D_real-ideal_  D_real-self_ | r= 0.148  r= 0.162  r= 0.136 | r= 0.136  r= 0.173*  r= 0.187* |
| 6^th^ semester | D_self-ideal_  D_real-ideal_  D_real-self_ | r= 0.328**  r= 0.270**  r= 0.202* | r= 0.380**  r= 0.112  r= 0.193* |
| 9^th^ semester | D_self-ideal_  D_real-ideal_  D_real-self_ | r= 0.253**  r= 0.08  r= 0.180* | r= 0.396**  r= 0.024  r= 0.114 |
| Final year | D_self-ideal_  D_real-ideal_  D_real-self_ | r= 0.231**  r= 0.028  r= 0.092 | r= 0.245**  r= 0.031  r= 0.099 |
| Total sample | D_self-ideal_  D_real-ideal_  D_real-self_ | r= 0.242**  r= 0.113**  r= 0.138** | r= 0.309**  r= 0.081*  r= 0.142** |

^*^Significant; ^**^Highly significant;
